# Accidental Fish Bone Ingestion Leading to Gastric Perforation: A Diagnostic Challenge

**DOI:** 10.7759/cureus.79025

**Published:** 2025-02-14

**Authors:** Panuwat Pornkul, Renae Bertucci, Nicole Hawkins, Sabin Smith

**Affiliations:** 1 Department of Surgery, Townsville University Hospital, Townsville, AUS; 2 Department of General Surgery, Cairns Private Hospital, Cairns, AUS

**Keywords:** acute abdomen, acute surgical abdomen, fish bone, fish bone perforation, gastric injury, perforated stomach, perforated viscera, rare cause of acute abdominal pain

## Abstract

Accidental ingestion of animal bones, including fish bones, often goes unnoticed and rarely leads to acute medical complications. However, in rare cases, fish bones can perforate the gastrointestinal tract, causing serious complications such as perforation, abscess formation, or fistula development, necessitating emergent surgical intervention. This case report describes a rare case of gastric perforation by a fish bone ingestion, complicated by perigastric and hepatic abscesses. The patient initially presented to a rural emergency department with acute abdominal pain, where limited imaging resources posed a diagnostic challenge. After repeated rural emergency department presentations, a definitive diagnosis was only established after the patient was transferred to a tertiary center and evaluated using computed tomography (CT) imaging. This helped to guide appropriate surgical management, and an emergency laparotomy was performed, which was followed by an uneventful recovery. We emphasize the critical role of CT imaging in diagnosing fish bone perforation, discuss common sites of perforation, and highlight the need to consider this pathology as a differential diagnosis in patients with undifferentiated abdominal pain.

## Introduction

Accidental ingestion of animal bones, particularly fish bones, is common but typically results in uneventful passage through the gastrointestinal (GI) tract. Most ingested fish bones are eliminated without symptoms within one week. However, in rare cases, the sharp edges of fish bones can perforate the GI tract, leading to complications such as mucosal injury, perforation, abscess formation, fistula development, or intestinal obstruction [1]. The overall incidence of GI perforation due to foreign body ingestion is estimated to be less than 1% [2]. Fish bones are a well-documented cause, yet they are often under-recognized due to their rarity, small size, delayed clinical presentation, and the absence of a clear history. Most patients do not recall ingesting a fish bone.

Perforation can occur anywhere along the GI tract but is most commonly observed at anatomical points of acute angulation, such as the ileocecal junction, duodenum, or sigmoid colon. Diagnosing fish bone perforation poses a significant challenge, as patients often do not recall ingestion, and clinical presentation is typically nonspecific. Symptoms may range from mild inflammatory changes to severe complications such as abscess formation, peritonitis, obstruction, or GI bleeding, frequently mimicking other acute abdominal pathologies such as acute appendicitis or diverticulitis [3,4]. This diagnostic ambiguity often results in delayed recognition or misdiagnosis.

Preoperative identification of fish bone perforation is rare, with only 23% of cases confirmed before surgical intervention, despite advancements in imaging techniques [5]. While upper GI endoscopy, laparoscopy, or exploratory laparotomy often provide definitive management, the ability to establish an early diagnosis remains limited, particularly in rural settings where access to advanced imaging and specialist care may be restricted.

Despite the high prevalence of recreational fishing in the North Queensland region, this case report describes the first documented instance of gastric perforation by a fish bone in Australia. It highlights the challenges of diagnosing fish bone perforation, particularly in rural emergency settings, where limited imaging capabilities and nonspecific presentations further complicate timely intervention.

## Case presentation

A 63-year-old man was transferred from a rural hospital to a tertiary care center after presenting to the emergency department twice within a short period. His initial presentation involved severe central upper abdominal pain and right-sided inguinal pain. He was discharged after a normal groin ultrasound, unremarkable chest and abdominal X-rays, and effective analgesia administration. During his second presentation two days later, he reported worsening upper abdominal pain and anorexia, leading to his admission for observation. A clinical re-examination later in the afternoon revealed a rigid abdomen. These findings prompted his transfer to a tertiary center for further evaluation with computed tomography (CT) later that evening.

Upon arrival, the patient continued to report severe upper abdominal pain as his chief complaint. His surgical history included two prior presentations for rectal foreign body insertion and bilateral open inguinal hernia repairs, with no significant past medical history. The patient was a non-smoker and non-drinker and denied illicit drug use. On examination, he was afebrile, with all vital signs within normal limits. Abdominal examination revealed tenderness in the epigastric and umbilical regions without signs of peritonism. There was no recurrence of a hernia in either inguinal region.

**Figure 1:**
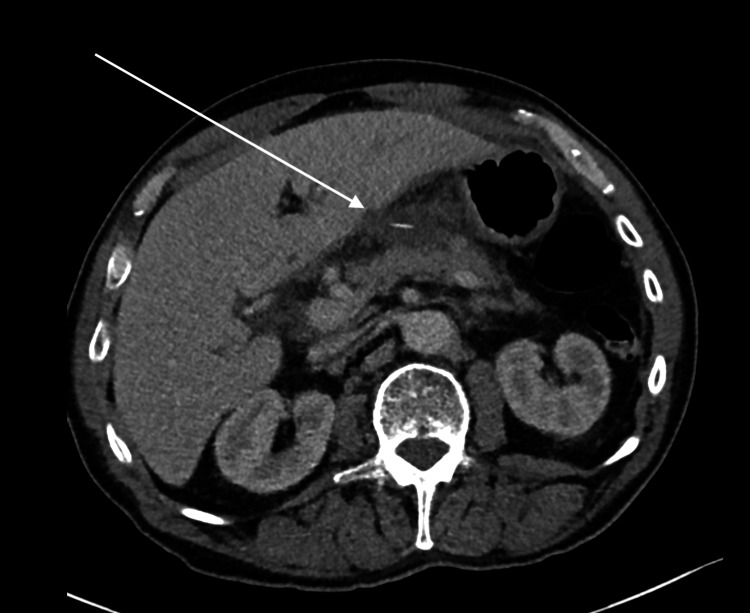
Computed tomography axial view of the upper abdomen. The arrow points to a hypodense collection within the lesser sac and a subcapsular hepatic collection measuring 25 × 38 × 33 mm (AP × TV × CC). At the center of this collection is a linear fish bone. AP, anteroposterior; TV, transverse; CC, craniocaudal

**Figure 2:**
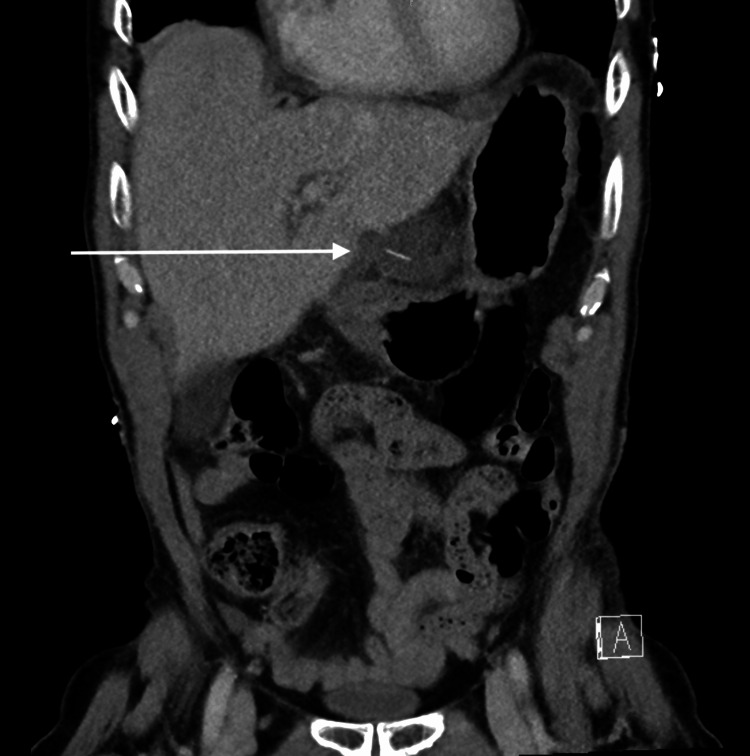
Computed tomography coronal view. The arrows point to a hypodense collection within the lesser sac, with the fish bone at the center appearing to be 25 mm away from the lesser curvature of the stomach.

Initial laboratory investigations at the tertiary emergency department revealed an elevated white cell count and inflammatory markers, suggesting a significant intra-abdominal infective process (Table 1). Intravenous contrast-enhanced CT of the abdomen and pelvis revealed a hypodense collection within the lesser sac and a subcapsular hepatic collection adjacent to segments 3 and 4b, both associated with a 15 mm radiodense linear object extending from the lesser curvature of the stomach (Figures 1-2). These findings were indicative of likely infective collections, supported by significant inflammatory stranding and local lymphadenopathy. Upon further questioning, the patient could not recall the last time he consumed fish, let alone ingested a fish bone.

**Table 1:** Admission blood tests on presentation to the tertiary emergency department. ALP, alkaline phosphatase; GGT, gamma-glutamyl transferase; ALT, alanine aminotransferase; AST, aspartate aminotransferase; CRP, C-reactive protein

Parameter	Result	Reference range (units)
Hemoglobin (g/L)	139	135-190
White cell count (x10^9^/L)	21.7	4-10
Platelet count (x10^9^/L)	314	140-400
Neutrophils (x10^9^/L)	19	2-8
Bilirubin (Total) (µmol/L)	21	<20
ALP (µmol/L)	104	30-110
GGT (µmol/L)	101	<55
ALT (µmol/L)	66	<45
AST (µmol/L)	19	<35
CRP (mg/L)	152	<5

An emergency exploratory laparotomy was performed the following day, with access to the lesser sac achieved through the hepatogastric ligament. An infective collection within the lesser sac was identified, discharging purulent material. An extraluminal fish bone was located within the cavity and subsequently removed. The abscess was drained, and no additional collections or defects were found in the lesser curvature or antrum of the stomach. The abdominal cavity was thoroughly irrigated, and the incision was closed. A total of four days elapsed from the initial presentation to the provision of definitive surgery.

Postoperatively, the patient’s recovery was uneventful. A CT scan with oral contrast conducted three days postoperatively showed no evidence of gastric leak. Two weeks after discharge, an upper endoscopy revealed chronic gastritis but no peptic ulcers or signs of previous perforation. The patient was discharged on proton pump inhibitor therapy for continued management.

## Discussion

Fish and chicken bones are the most commonly ingested bones that can cause GI perforation. Risk factors include pediatric age, intellectual impairment, denture use, consumption of unfilleted fish, and alcohol or substance misuse [2]. In retrospect, the patient had no recollection of recent fish consumption, further complicating the diagnostic process in this case.

GI perforation by fish bones can occur along the entire gastrointestinal tract, with perforation most commonly occurring in anatomical regions with significant angulation. A retrospective study of 62 patients by Goh et al. reported that 29% of perforations occurred extra-abdominally at the distal rectum or anus, while 71% were intra-abdominal, with the ileum and jejunum being the most common sites. Similarly, Rodriguez et al. studied 33 patients and found that 54.5% of perforations were extraperitoneal, primarily rectal, followed by the terminal ileum in 21.2% of cases [6,7]. Given these findings, fish bone perforation should be considered a differential diagnosis in cases of acute abdomen with uncertain origin, particularly when clinical history, physical examination, and investigative findings are inconsistent with more common surgical conditions [8]. Diagnosis can be further complicated in rural and regional hospital settings due to limited physician experience with uncommon surgical pathologies. Paradoxically, these communities in Australia are more likely to engage in recreational fishing and self-consumption, increasing the risk of accidental fish bone ingestion and subsequent complications.

Stomach perforation by fish bone is relatively rare. To date, no cases have been documented in Australia or New Zealand, with only a few reports internationally. Lee et al. described a case where a fish bone perforated the gastric antrum, leading to an abscess in the left lobe of the liver. Similarly, the patient presented with mild epigastric pain and intermittent fevers for over a week [9]. Delayed presentation in gastric, duodenal, and colonic perforations may occur due to the thicker walls of these GI segments, which require gradual penetration and migration of the foreign body. This process allows adjacent organs, such as the liver, to *seal off* the perforation through abscess formation. Retroperitoneal perforations can present more insidiously, as it may not invoke acute peritonitis [6].

An accurate preoperative diagnosis of foreign body perforation is crucial for planning the most appropriate and least invasive surgical intervention. However, diagnostic imaging of fish bones presents challenges. Plain radiography is often ineffective due to the variable radio-opacity of fish bones, and pneumoperitoneum may not reliably indicate small perforations. Ultrasonography can detect hyper-reflective foreign bodies and complications like abscesses, but its utility is limited by operator dependency, patient body habitus, and anatomical inaccessibility. The utility of usual imaging modalities are diminished when assessing fish bones compared to other commonly ingested foreign objects such as chicken bones, batteries, and coins [10]. CT is commonly used to evaluate acute and recurrent abdominal pain. Its sensitivity for detecting intra-abdominal fish bones is 71.4% at a slice thickness of 7-7.5 mm, increasing to 100% with retrospective review by a senior radiologist [11]. However, CT has limitations, including observer bias, obscuration by oral contrast, and the misinterpretation of fish bones as blood vessels in contrast-enhanced studies [10]. Despite these limitations, CT remains the preferred imaging modality due to its effectiveness, convenience, and ability to rule out other diagnoses.

## Conclusions

Fish bone perforation is a diagnostic challenge and an important cause of acute abdominal pain, often overlooked due to its rarity and nonspecific clinical presentation. While CT imaging is key for detection, limited imaging access in rural Australia can delay diagnosis. This case exemplifies one of the many ways fish bone perforation may present and underscores the importance of considering this as a differential diagnosis in patients with unexplained abdominal pain, particularly in populations where fish is consumed. Timely transfer to a tertiary center enabled diagnosis and definitive clinical management, leading to successful surgical intervention and recovery.
